# The Leptin Receptor Complex: Heavier Than Expected?

**DOI:** 10.3389/fendo.2017.00030

**Published:** 2017-02-21

**Authors:** Joris Wauman, Lennart Zabeau, Jan Tavernier

**Affiliations:** ^1^Cytokine Receptor Laboratory, Faculty of Medicine and Health Sciences, Department of Biochemistry, Ghent University, Ghent, Belgium; ^2^VIB Medical Biotechnology Center, VIB, Ghent, Belgium

**Keywords:** leptin, leptin receptor, activation, signaling, cross-talk, leptin resistance

## Abstract

Under normal physiological conditions, leptin and the leptin receptor (ObR) regulate the body weight by balancing food intake and energy expenditure. However, this adipocyte-derived hormone also directs peripheral processes, including immunity, reproduction, and bone metabolism. Leptin, therefore, can act as a metabolic switch connecting the body’s nutritional status to high energy consuming processes. We provide an extensive overview of current structural insights on the leptin–ObR interface and ObR activation, coupling to signaling pathways and their negative regulation, and leptin functioning under normal and pathophysiological conditions (obesity, autoimmunity, cancer, … ). We also discuss possible cross-talk with other receptor systems on the receptor (extracellular) and signaling cascade (intracellular) levels.

## Introduction

The identification of two spontaneous obese mouse strains at the Jackson Laboratory, *ob/ob* mice in 1950 and *db/db* mice in 1965, were the first steps toward the discovery of the leptin–ObR system. A series of elegant parabiosis experiments illustrated that *db/db* mice overexpressed a strong circulating satiety factor to which they cannot respond themselves. On the other hand, *ob/ob* animals do not produce this factor but lose weight when parabiotically paired to wild-type or *db/db* mice (1). This factor was cloned 40 years later by Friedman and colleagues as the product of the *ob* gene and called leptin after the Greek “leptos” meaning thin (2). The product of the *db* gene was identified as the ObR using an expression-cloning strategy based on the ability to bind leptin (3).

## Leptin

Leptin, a hormone with cytokine-like characteristics, is mainly but not exclusively produced by adipose tissue in a way that its levels positively correlate with the energy stored in the body (4–6). Other sources of (lower) leptin expression include placenta, stomach, mammary epithelium, and skeletal muscle (7–9). Mature leptin is a non-glycosylated 16 kDa protein of 146 amino acids. The crystal structure at 2.4 Å resolution of leptin W110E, a mutation that dramatically increases solubility of the protein without affecting biological activity, shows a typical four-helical bundle structure. Four anti-parallel α-helices (A, B, C, and D) in an up-up-down-down arrangement are connected by one short (BC) and two long (AB and CD) loops. Leptin has two conserved cysteine residues (one in the CD loop and the C-terminal residue) that form a solvent-exposed disulfide bridge that tethers the CD loop to the C-terminal part of helix D. This disulfide bridge is crucial for structural stability, secretion, and biological activity (10, 11). These structural characteristics strongly resemble those found in granulocyte-colony stimulating factor (G-CSF) and interleukin-6 (IL-6) cytokines, and leptin is, therefore, classified as a long-chain cytokine.

## Leptin Receptor

ObR is a single membrane-spanning receptor belonging to the class I cytokine receptor family (3). Up to now, six ObR isoforms are produced by alternative splicing or ectodomain shedding: ObRa–ObRf. These include one long form (ObRb; with an intracellular domain of 302 Aa); four short forms (ObRa, ObRc, ObRd, and ObRf, with cytoplasmic tails of 30–40 Aa with unique C-termini); and one soluble form (ObRe). ObRb contains three highly conserved tyrosine residues (Y985, Y1077, Y1138) required for efficient leptin signaling. ObRb is highly expressed in specific nuclei of the hypothalamus, a region of the brain involved in the regulation of body weight (12–14). Expression at functional levels can also be detected in a broad range of other cell types, in line with the pleiotropic effects of leptin (see further). Their expression pattern suggests that the short ObR’s play a role in transport of leptin over the blood–brain barrier (BBB) (15) and/or renal clearance (3). The soluble ObRe isoform, directly secreted in mice while in humans generated by ectodomain shedding (16, 17), modulates bio-availability of the hormone (18). All isoforms have an identical extracellular part consisting of six domains: an N-terminal domain (NTD), two CRH domains (CRH1 and CRH2), an immunoglobulin-like domain (IGD), and two additional membrane-proximal fibronectin type III (FN III) domains. This overall architecture of the extracellular domain and the sequence similarity resemble that of the G-CSF and gp130-related receptors. The ObR is heavily glycosylated causing an increase of 30–70 kDa in molecular weight (19, 20). N-glycosylation is predominant with 18 of 20 NXS motifs in the human receptor glycosylated, although some O-glycosylation is also present (19). *N*-glycanase F treatment reduces leptin binding of recombinant ObR extracellular domain by 80%, illustrating the importance of this type of modification in this process (20).

## Leptin Biology and Disease

As mentioned above, loss-of-function mutations in the leptin or ObR genes (2, 21–23) or genetic ablation of leptin’s central signaling (16, 24) results in severe, early-onset obesity (25). To date, leptin and its receptor are the most crucial of factors identified, which control body weight by balancing food intake and energy expenditure in the adaptive response to altered energy states like fasting or starvation. As a product of adipose tissue, it signals the energy stored in the body and thereby functions as a negative feedback adipostat, an efferent satiety signal and an anti-obesity hormone.

In 2014, more than 1.9 billion adults were overweight (BMI levels 25–29 kg/m^2^), and of these, over 600 million were obese (BMI levels 30 kg/m^2^ and greater), making obesity and the associated metabolic syndrome a major health problem worldwide. It is a complex medical condition caused by an accumulation of excess body fat (calorie intake exceeds calorie expenditure) leading to negative effects on health including type 2 diabetes, heart diseases, obstructive sleep apnea, cancer, and joint disease. Obesity is caused by the interplay between environmental, genetic, and epigenetic factors. To date, not less than 50 genes (including leptin, ObR, and mediators of leptin signaling) are related to an increased obesity risk in humans and rodents (26). Paradoxically, levels of biologically active leptin are elevated in most obese subjects, and these patients do not respond to leptin treatment. Mechanisms underlying this “leptin resistance” are discussed later.

Leptin or ObR deficiencies not only cause severe obesity but also abnormalities in hematopoiesis (27), immunity (28), reproduction (29), angiogenesis (30), bone formation (31), and blood pressure (BP) (32). This lead to the concept that leptin can act as a “metabolic switch” that links the body’s energy stores to these high-energy demanding processes (33).

Ozata and colleagues were the first to report that seven members of a Turkish family with congenital leptin deficiency died during childhood due to infections pointing to a role for leptin in human immunity (34). Along the same line, leptin treatment of two children with congenital leptin deficiency (35) and in females with acquired hypoleptinemia (36) normalized absolute T cell numbers and nearly restored T cell proliferation responses and cytokine release profiles. Over the past decade, leptin emerged as a regulator of multiple cell types of both innate and adaptive immune responses (37). In innate immunity, leptin controls the activation of macrophages, neutrophils, monocytes, dendritic, and natural killer cells and promotes the production of pro-inflammatory cytokines. Thymic and splenic homeostasis, naïve CD4^+^ cell proliferation, promotion of T_H_1 responses, suppression of regulatory T cells, and activation of T_H_17 cells are the most important functions in adaptive immunity (37). Its pro-inflammatory characteristics link leptin to the onset and progression of several autoimmune diseases, including multiple sclerosis (38), antigen-induced arthritis (39), hepatitis (40, 41), colitis (41), and glomerulonephritis (42). Leptin-deficient rodents are often protected in experimental models for these diseases, while leptin administration restores sensitivity.

The observations that humans and rodents with congenital leptin deficiencies are sterile and that anorexia and obesity delay and accelerate the onset of puberty, respectively, led to the idea that leptin is an important player in reproduction (43). The hormone functions both directly on the ovaries and indirectly *via* gonadotrophin-releasing hormone, luteinizing hormone, or kisspeptin release (44).

*Ob/ob* and *db/db* mice display significant longer vertebral length and have higher bone mass (31). Leptin influences bone metabolism *via* central and peripheral pathways [reviewed in Ref. (45)]: it suppresses osteoblast proliferation and promotes osteoclast resorption through activation of neurons in the ventromedial hypothalamus. More direct effects include proliferation, survival, differentiation, or suppression of bone marrow mesenchymal stem cells, osteoblasts, osteoclasts, and chondrocytes, and the synthesis of collagen and extracellular matrix proteins.

Leptin affects BP in an apparently opposite way: on the one hand it causes chronic increase in BP and may contribute to obesity related hypertension, while on the other hand its metabolic actions (lowering appetite and increasing energy expenditure) tend to reduce BP (46).

Excess body fat in obesity can contribute to the development of cancer. Etiological causes not only include elevation of estrogen, insulin, insulin-like growth factors, leptin but also local inflammation and depressed immune function seen with excess adiposity. Elevated leptin levels are linked to an increased risk of myeloma (47) and of prostate (48), breast (49), colorectal (50), and renal (51) cancers. Leptin not only promotes survival and proliferation of several cancer cell lines directly but also promotes adhesion, invasion, metastasis (*via* upregulation of metalloproteinases, E-cadherin, and extracellular matrix proteins), and angiogenesis (*via* vascular endothelial growth factor and its receptor) in the tumor environment (52).

## ObR Activation

Only a minor fraction of the ObR (10–20%) is expressed at the cellular surface, while the majority is found in intracellular compartments, including endoplasmic reticulum (ER), *trans*-Golgi, and endosomes (53). This subcellular distribution is the result of the partial retention of the receptor in the biosynthesis pathway and the constitutive, ligand-independent endocytosis of the receptor (see also further). Furthermore, co-immunoprecipitation of differentially tagged ObR’s (54, 55) or the high basal signal in the absence of leptin in bioluminescence resonance energy transfer (BRET) (56) and fluorescence resonance energy transfer (FRET) (57) suggests that the ObR forms pre-formed dimers (or oligomers) on the cellular surface. Numerous cytokine receptors can form these inactive, pre-formed receptor complexes, including the erythropoietin receptor (58–60), the growth hormone receptor (61), and the IL-6 receptor (62). Earlier studies suggested this oligomerization also in solution (63, 64), but more recent multi-angle laser light scattering and small-angle X-ray scattering (SAXS) show that the soluble ObRe is a monomer in the absence of leptin (65, 66).

**Figure 1:**
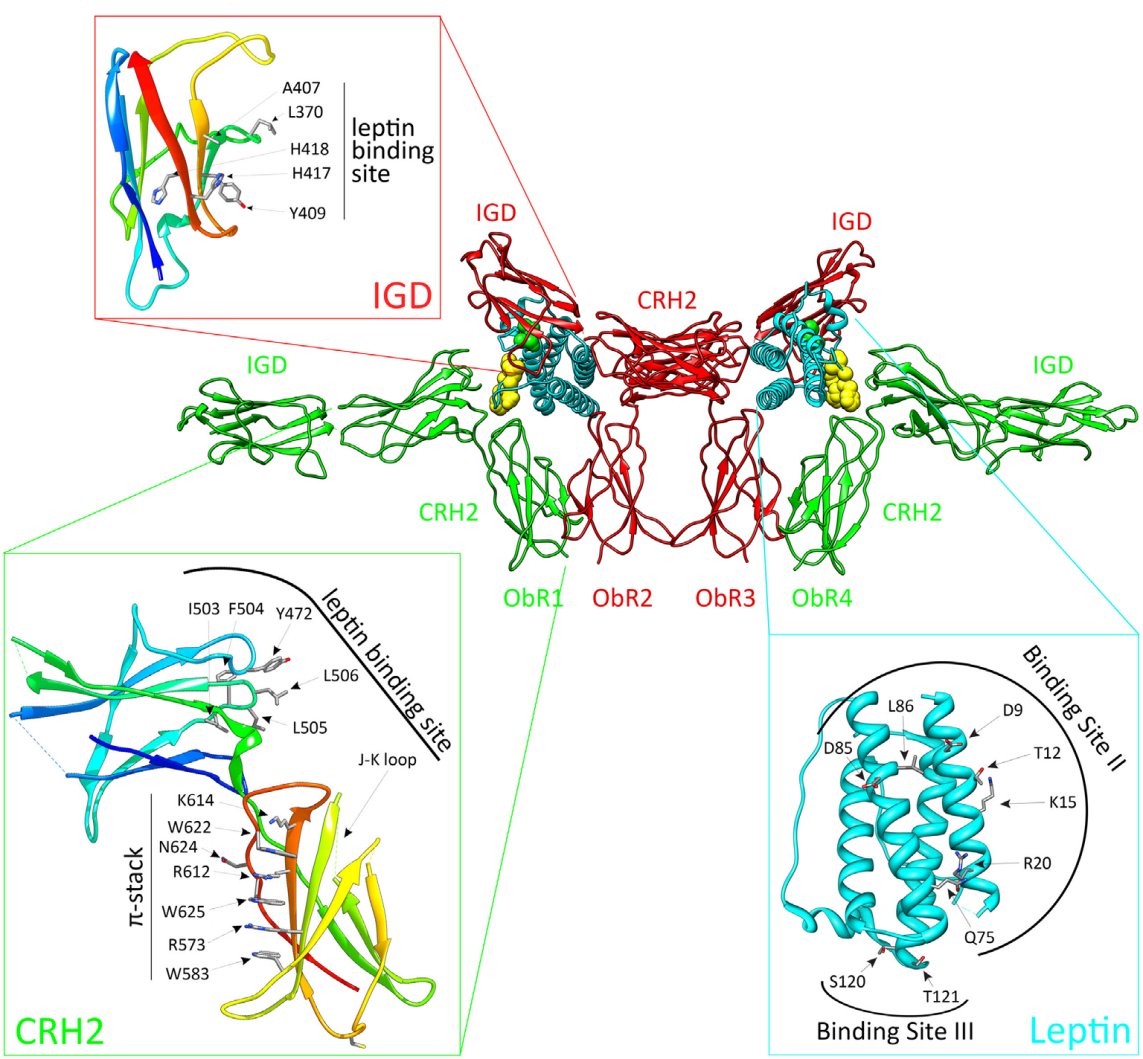
**The activated leptin:ObR complex**. Leptin clusters two pre-formed ObR dimers to form an activated 2:4 leptin:ObR complex. In this model, the ObR’s are colored green and red, leptin molecules cyan. The hormone binds with its binding site II to the CRH2 domain of the receptor, while site III residues interact with the immunoglobulin-like domain (IGD) of a second receptor. These residues are colored yellow and green, and defined in the cyan panel. Receptor residues involved in these interactions are shown in the red and green panels. For reasons of clarity, only the CRH2 and IGD domains of each receptor are shown.

ObR activation depends on the CRH2, IGD, and FN III domains (Figure 1). The ObR CRH2 domain is the major leptin-binding determinant in the receptor (67, 68). Depending on the technique and proteins used, CRH2 binds leptin with a *K_D_* of 0.2–15 nM in solution or 0.2–1.5 nM on the cellular surface [reviewed in Ref. (69)]. The binding affinity to the isolated CRH2 domain or to the complete extracellular domain is comparable, illustrating that this domain is indeed strictly required and sufficient (67, 70). A region of four consecutive hydrophobic residues has been identified as the leptin-binding site in the CRH2 domain (71, 72). The crystal structure of the CRH2 domain, in complex with a Fab fragment of a neutralizing Ab, was determined at 1.95 Å resolution (73). This is the first and, hitherto, the only high-resolution structure for (a part of) the ObR. The IGD and membrane-proximal domains have no detectable affinity for the ligand, but are nonetheless indispensable for receptor activation. Deletion of the IGD results in a receptor with wild-type affinity for leptin, but completely devoid of biological activity (67, 68). A conserved surface patch in the β-sheet formed by β-strands 3, 6, and 7 was identified as the leptin-binding site in this domain (74). Leptin contains a binding site (III) which allows contact with the IGD of a second ObR, thereby inducing dimerization and potential higher-order clustering with evidence for a 2:4 leptin:ObR hexameric structure (74). In the FN III domains, two conserved cysteine are crucial for receptor activation since combined mutation completely blocks activation of the receptor (64). Finally, deletion of the NTD and CRH1 domains hardly affects ObR functionality (67, 68). This seems in contrast to the obese phenotype of fatty Zucker rats resulting from the Q269P mutation in the CRH1 domain (75). Likewise, the naturally occurring single-nucleotide polymorphism Q223R causes obesity in Brazilian multiethnic subjects (76) and the increased susceptibility toward protozoan infections in children (77).

Based on the structural and evolutionary relationship of leptin and its receptor with the G-CSF and IL-6 receptor systems, we selected and tested a panel of leptin mutants and thereby identified three putative receptor-binding sites (78). Mutations in binding site I (located at the C terminus of helix D) moderately affect binding and signaling. Binding site II residues (at the surface of helices A and C) are crucial for binding to the CRH2 domain, but mutations in this region have only limited effect on signaling (78). Two regions were independently proposed as the binding-site III, which interacts with the ObR IGD: the area around residues S120 and T121 at the N terminus of helix D (78) and the 39-LDFL-42 stretch in the AB loop (79). Mutation of either area is sufficient to create leptin antagonists *in vitro* and *in vivo* (78, 79).

Both 3D reconstructions of 2D negative-stain EM images by Mancour et al. (65) and the SAXS experiments by Moharana et al. (66) point to an analog 2:2 quaternary core leptin:ObR complex. In this model, leptin binds with its site II to the CRH2 domain of a first ObR and engages a second receptor *via* the site III–IGD interaction. Additional receptor–receptor interactions (e.g., between FN III domains) that occur at the cell surface, but not in solution, could lead to 2:4 or 4:4 leptin:ObR complexes. This higher-order clustering would be in line with our signaling-complementation assay (68). Finally, leptin stimulation increases BRET and FRET signals (see above), suggesting that reorganization within the pre-formed complexes and/or *de novo* oligomerization likely occur (56, 57).

## ObR Signaling

**Figure 2:**
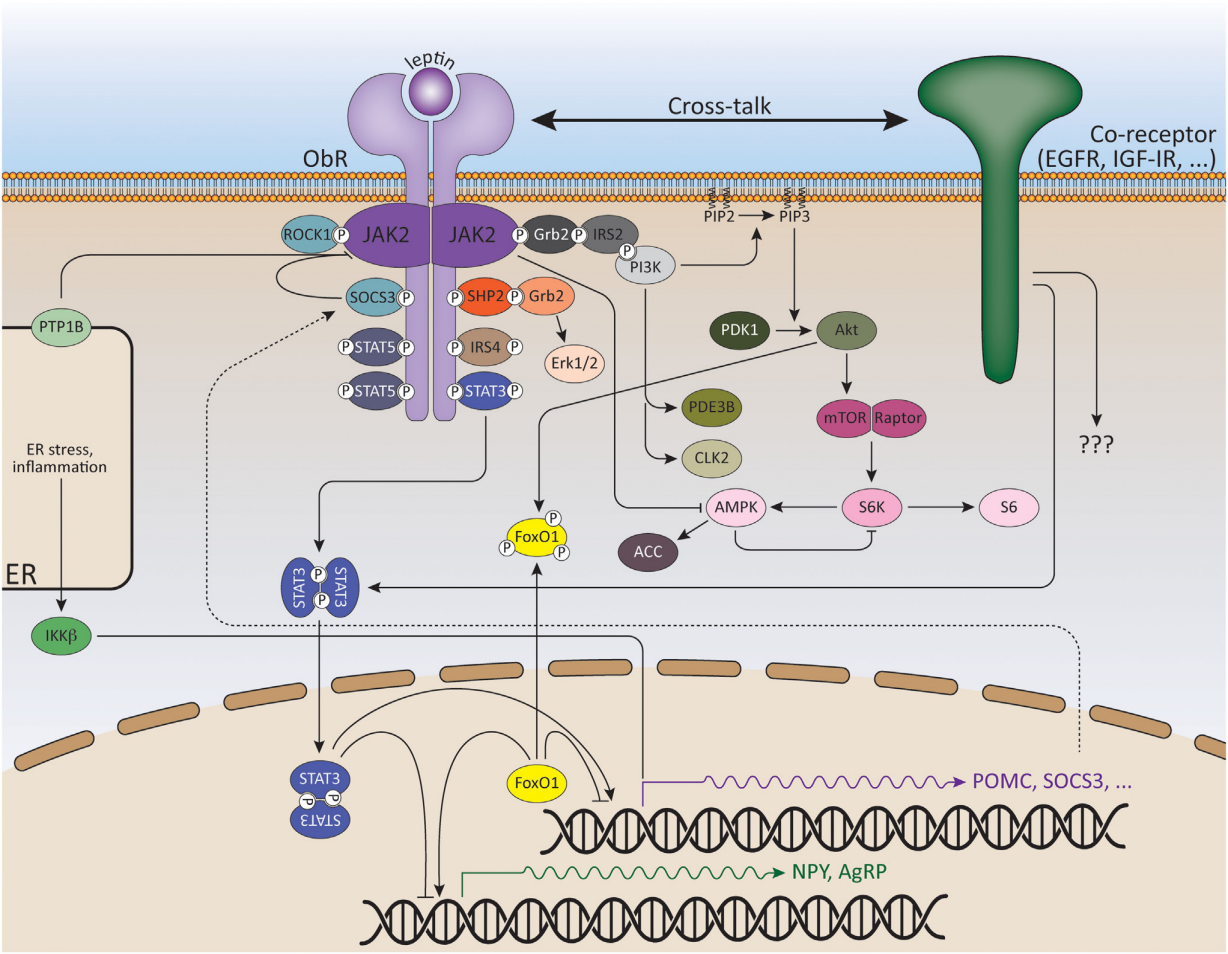
**Signaling pathways of leptin and its downstream effectors**. ObR oligomerization (here only dimerization shown for reasons of clarity) results in phosphorylation and activation of cytoplasmic associated JAK2 kinases. These activated JAKs phosphorylate tyrosine residues in the cytoplasmic tail of the receptor. Recruitment and activation of secondary signaling molecules allow ObR signaling *via* the JAK/STAT, MAPK, PI3K, AMPK, and mTOR pathways. See text for more details.

The ObRb is mainly expressed by two distinct neuronal populations in the hypothalamic arcuate nucleus (ARC): the anorexigenic (appetite-depressing) POMC (pro-opiomelanocortin) neurons and the orexigenic (appetite-promoting) NPY/AgRP (neuropeptide Y/agouti-related peptide) neurons (80, 81) (Figure 2). ObRb activation in POMC neurons triggers POMC expression, a precursor peptide, that is further converted by prohormone convertases to α-melanocyte stimulating hormone (αMSH), which is secreted and signals by activating melanocortin receptors, MC3R, and MC4R. Deletion of MC3R and/or MC4R results in leptin resistance and obesity (82, 83). Specific reconstitution of ObRb expression in POMC neurons of ObRb-deficient mice not only modestly reduces body weight but also completely normalizes blood glucose levels, insulin sensitivity, and locomotor activity, indicating that leptin signaling in POMC neurons has a key role in regulating glucose homeostasis (84, 85). In contrast to POMC neurons, leptin suppresses the activity of NPY/AgRP neurons and the associated secretion of its orexigenic neuropeptides. AgRP is a potent antagonist of αMSH. Moreover, NPY/AgRP neurons innervate POMC neurons and inhibit POMC neuronal firing by releasing GABA (γ-aminobutyric acid) (86, 87). Selective deletion of ObRb in POMC or NPY/AgRP neurons only gives rise to a mild obese phenotype indicating that also other regions in the brain are involved in the control of energy homeostasis by leptin (88–90).

### The JAK/STAT Pathway

The JAK/STAT (Janus kinase/Signal transducers and activators of transcription) pathway is probably the best explored pathway activated by leptin. Upon ligand binding and JAK2 activation, the conserved ObRb phosphotyrosine 1138 motif serves as a binding site for the SH2 domain of STAT3. STAT3 itself will subsequently become phosphorylated by the JAKs on Y705 and translocate as dimers to the nucleus, where it will modulate expression of several STAT3-responsive target genes, such as Suppressor of cytokine signaling 3 (SOCS3) (91, 92). SOCS3 acts as a potent negative regulator of the JAK/STAT pathway, thereby forming a negative feedback loop (93). The negative regulation of ObR signaling will be further discussed in the following section.

Experiments with neuron-specific STAT3^−/−^ mice have pinpointed the essential role of STAT3 for the acute anorectic actions of leptin (94–96). Mice in which STAT3 is specifically deleted in ObRb neurons similary develop hyperphagic obesity with some preservation of glucose homeostasis (97). However, despite the fact that the POMC promotor has a STAT3-responsive element and that deletion of STAT3 in POMC neurons completely diminishes POMC induction, POMC-specific STAT3 knock-down did not completely abrogate the anorexigenic effect of leptin and only leads to a mild obese phenotype (98, 99). This indicates that also other signaling pathways in POMC neurons are involved in the energy homeostatic response to leptin and/or STAT3-dependent effects in other, non-ARC cells are involved. Likewise, genetic inactivation of STAT3 in NPY/AgRP neurons leads to mild hyperphagia, a decreased response to leptin and increased levels of basal NPY expression, while AgRP expression remains unaltered (100, 101).

Substitution of tyrosine 1138 with serine (*s/s* mice) disrupts STAT3 recruitment to the ObRb. *S/s* mice are, therefore, hyperphagic and obese, similar to *db/db* mice and show a suppression of melanocortin activity. However, *s/s* mice are less hyperglycemic and show normal fertility, indicating that STAT3-independent pathways control leptins’ effects on reproduction and glucose metabolism (102–104).

In addition to phosphorylation, leptin-induced STAT3 methylation by protein arginine *N*-methyltransferase 2 (PRMT2) was also shown to occur, and PRMT2^−/−^ mice are lean, hypophagic, have reduced serum leptin levels and are more resistant to diet-induced obesity (DIO) compared to wild-type littermates (105). More recently, it was demonstrated that the nuclear receptor Nur77 (or TR3) facilitates STAT3 acetylation by recruiting acetylase p300 to and dissociating deacetylase HDAC1 (histone deacetylase 1) from STAT3, thereby enhancing the transcriptional activity of STAT3 and the expression of POMC (106).

Next to STAT3, leptin also activates STAT1, STAT5, and STAT6 in cell culture (107, 108). However, only leptin-dependent hypothalamic STAT5 phosphorylation was observed *in vivo* (109, 110). Activated STAT5 binds to pY1077, and to lesser extent pY1138 of the ObRb (111, 112). ObRb Y1077 mutants show only mildly increased food intake and adiposity. Furthermore, female mice display impairments in estrous cycling, suggesting that signaling by ObRb Y1077 plays only a modest role in the control of metabolism by leptin, while it may link body adiposity to the reproductive axis (113). However, deletion of STAT5 in ObRb expressing cells has no effect on body weight and fertility (114), indicating that neither STAT3 nor STAT5 are required for the regulation of fertility by leptin and that other signaling pathways depending on Y1077 might be involved in the leptin’s reproductive functions.

### The MAPK Pathway

As mentioned before, the ObRb has three conserved tyrosine residues of which the pY985 motif serves as a docking site for the C-terminal SH2 domain of SHP2 (SH2-containing protein tyrosine phosphatase 2) (115–117). On its turn, SHP2 will become phosphorylated by the JAKs and recruit the adaptor protein Grb2 (growth factor receptor-bound protein 2), which ultimately leads to the activation of the mitogen-activated protein kinase (MAPK) extracellular signal-regulated kinase 1/2 (ERK1/2) (115, 118, 119). The phosphatase activity of SHP2 is necessary for leptin-mediated ERK activation (115). Weaker ERK activation by the short isoform ObRa (independent of Y985) was also shown, most likely *via* direct binding of Grb2 to JAK2 (120).

Neuron-specific deletion of SHP2 or pharmacological inhibition of ERK1/2 in the hypothalamus blocks the anorectic effects of leptin (118, 119). Furthermore, POMC-specific deletion of SHP2 results in mild obesity and increased susceptibility to DIO (121, 122), while female mice expressing a constitutively active form of SHP2 in the brain are resistant to DIO (123), supporting the role for SHP2 in the control of energy homeostasis. However, since SHP2 participates in many signaling pathways, the specific contribution of SHP2 downstream the ObRb remains difficult to assess. Mice with a mutation in Y985 are neuroendocrinologically normal and fertile, but especially female mice demonstrate decreased hypothalamic AgRP expression, increased pSTAT3 levels and leptin sensitivity, and resistance to DIO (124). Young homozygous Y985F mice were shown to be slightly leaner, although they exhibit adult-onset obesity (125). These data most likely do not reflect the role of Y985 in leptin-dependent ERK activation, but are consistent with increased ObRb signaling due to decreased feedback inhibition *via* disruption of SOCS3 binding (see further).

### The PI3K–Akt–Foxo1 Pathway

A crucial role for the PI3K (phosphatidylinositol 3-OH kinase) pathway in leptin signaling was first demonstrated when intracerebroventricular (ICV) injection with PI3K inhibitors inhibited leptin’s anorexigenic effects (126). However, the activation mechanism is difficult to dissect since no ObRb phoshotyrosine site has been identified that mediates PI3K activation. Also, the PI3K pathway is shared with other receptors, especially the insulin receptor (IR). This hinders the evaluation of the ObRb-specific contribution of PI3K in the control of energy homeostasis. Although there is evidence that PI3K activation mediates the acute effects of leptin on the neuroelectrical activity of POMC cells and that the acute responses to leptin and insulin are largely segregated in distinct POMC subpopulations (127, 128). Nevertheless, mice with impaired PI3K signaling in POMC neurons have normal body weight, indicating that the PI3K pathway in these neurons is not critical for the leptin-mediated regulation of energy homeostasis (128).

Insulin receptor substrate 1, and particularly IRS2 are recruited to the ObRb *via* SH2B1, which interacts and upregulates the kinase activity of JAK2 (129, 130). SH2B1^−/−^ mice display hyperphagia and severe early-onset obesity (131). Neuron-specific restauration of SH2B1 rescues this phenotype and improves leptin-dependent signaling and neuropeptide expression in the hypothalamus (132, 133). Alternatively, IRS4 was also shown to recruit the PI3K regulatory p85 subunit *via* the Y1077 motif of the ObRb (134).

IRS proteins in turn bind the PI3K p85 subunit, leading to PI3K activation and the accumulation of PIP_3_ (phosphatidylinositol 3,4,5-triphosphate). This leads to the sequential activation of PDK1 (3-phosphoinositide-dependent protein kinase 1) and Akt, resulting in the inhibition of the transcription factor Foxo1 (Forkhead Box O1). Foxo1 mediates the anorectic effects of leptin by regulating the expression of POMC, AgRP, and NPY (135–138). Foxo1 (when activated) stimulates the transcription of AgRP and NPY, but suppresses the transcription of POMC; thereby antagonizing the transcriptional action of STAT3 in these hypothalamic subpopulations. Recently, it was shown that direct interaction of Foxo1 to STAT3 was responsible for the inhibition of STAT3-mediated leptin signaling (137, 139). Therefore, mice with POMC-specific PDK1 or Foxo1 depletion are less or more sensitive, respectively, to the anorectic effects of leptin (140, 141). Likewise, Foxo1 ablation in AgRP neurons results in reduced food intake, improved glucose homeostasis, and increased sensitivity to leptin (142).

As mentioned above, PI3K activity leads to PIP_3_ accumulation. PTEN (tumor suppressor phosphatase and tensin homology) promotes the opposite reaction *via* its lipid phosphatase activity. PTEN ablation in ObRb-expressing neurons induces enhanced PI3K activity and reduced body fat (143). On the other hand, POMC-specific PTEN^−/−^ mice develop leptin resistance and obesity, suggesting that chronic elevation of PIP_3_ in POMC neurons may interfere with hypothalamic leptin activity (144).

Another target activated downstream of PI3K/Akt is mTOR (mammalian target of rapamycin), an evolutionally conserved serine/threonine kinase, which senses nutrient availability and stimulates protein synthesis, cell growth, and proliferation. Leptin stimulates the phosphorylation of p70 S6 kinase (S6K) *via* mTOR. Selective inhibition/deletion of mTOR or S6K in the hypothalamus attenuates leptin’s anorexigenic effects (145–147). Additionally, PDE3B (phosphodiesterase 3B) is expressed in ObRb-expressing neurons in the hypothalamus (148). Leptin induces PDE3B activity, which results in a decrease in cAMP levels *via* the PI3K pathway. Inhibition of PDE3B activity by cilostamide reverses leptin’s effects on food intake and body weight, as well as the leptin-induced increase in POMC expression in the hypothalamus (149, 150).

### The AMPK Pathway

Like mTOR, adenosine monophosphate-activated protein kinase (AMPK) serves as an intracellular fuel sensor and is activated by elevated AMP/ATP ratios. AMPK is a heterotrimeric serine/threonine kinase consisting of a catalytic α subunit and regulatory β and γ subunits. Leptin regulates AMPK activity in a tissue-specific way: leptin activates AMPK in hepatocytes and muscle tissue (151, 152), while in the hypothalamus, leptin inhibits AMPK, hence reducing food intake and body weight (153, 154). A reduction in AMPK phosphorylation (at T172), observed upon leptin treatment, is followed by decreased phosphorylation and increased activation of the AMPK target, acetyl-CoA carboxylase (ACC), a key enzyme in fatty acid biosynthesis (155, 156). Dominant negative AMPK expression in the hypothalamus is sufficient to reduce food intake and body weight, while constitutively active AMPK attenuates leptin’s anorexigenic effects (153, 155).

The precise mechanism of leptin-mediated AMPK activation is still unclear but requires JAK2 activity and does not seem to depend on intracellular phosphotyrosine motifs in the ObRb (152). Of the two catalytic subunits, α1 and α2, leptin predominantly appears to modulate AMPK activity *via* alternative phosphorylation sites in the α2 subunit (153, 157). In the hypothalamus, leptin administration increases α2-AMPK phosphorylation at S491, which decreases AMPK activity. Mice lacking α2-AMPK in POMC neurons become obese due to dysregulated food intake and decreased energy expenditure (most likely *via* altered glucose sensing), while deletion of α2-AMPK in NPY/AgRP neurons results in an age-dependent lean phenotype (154). S6K was shown to form a complex with α2-AMPK, resulting in phosphorylation on S491 (158). Blocking α2-AMPK S491 phosphorylation increases hypothalamic AMPK activity, food intake, and body weight (158). Thus, mTOR-S6K signaling serves as an important signaling pathway upstream of AMPK in hypothalamic leptin signaling.

### Other ObRb Signaling Pathways

Mice with replacement of all three ObRb tyrosines with phenylalanines (ObRb^3F^) are slightly less obese than *db/db* mice and show significantly ameliorated glycemic control and fertility, illustrating that ObRb exerts crucial metabolic actions not only through ObR tyrosine-dependent but also ObR tyrosine-independent mechanisms (159). Interestingly, mice expressing a truncated ObRb mutant (ObRb^∆65c^), that retains JAK2 activity but lacks all ObRb tyrosines, have a similar phenotype as *db/db* mice showing obesity, diabetes, and infertility (160). Thus, the improved phenotype of ObRb^3F^ mice compared to ObR^∆65c^ mice reveals that JAK2-autonomous signaling is not sufficient to mediate these improvements. These data suggest the existence of non-canonical signaling pathways that may emanate from an uncharacterized ObRb site, independent of phosphorylation.

During the last few years, a role for several kinases in hypothalamic leptin signaling was proposed. Rho-kinase 1 (ROCK1) regulates leptin’s effect on body weight homeostasis by binding and activating JAK2. ROCK1 increases JAK2 phosphorylation and downstream activation of STAT3 and Foxo1. Mice lacking ROCK1 in either POMC or NPY/AgRP neurons, display impaired leptin sensitivity and obesity (161). A role for the RIIβ regulatory subunit of PKA (cyclic AMP-dependent protein kinase A) in modulating the magnitude and duration of ObRb signaling was also demonstrated (162). Mice lacking this PKA subunit display reduced adiposity and resistance to DIO. Recently, Cdc2-like kinase 2 (CLK2) activity was proven to be regulated by leptin in a PI3K-dependent manner and reduction of CLK2 expression in the hypothalamus was sufficient to abolish the anorexigenic effect of leptin (163).

## Leptin Resistance: Possible Mechanisms

The discovery of leptin in 1994 generated high expectations for its potential use as a therapeutic to combat obesity. However, obese individuals were found to be refractory to leptin therapy (164, 165). In fact, obesity is commonly accompanied with elevated circulating leptin levels (hyperleptinemia) in proportion to the increased body fat mass. This paradox is referred to as leptin resistance and the underlying mechanism is multifactorial: impairment in ObRb signaling, hypothalamic neuronal wiring, leptin transport into the brain and ObR trafficking, ER stress and inflammation (16).

### Negative Regulation of ObR Signaling

Leptin signaling is negatively regulated by SOCS3 and phosphatases, such as protein tyrosine phosphatase 1B (PTP1B), T-cell PTP (TCPTP), and RPTPϵ. Their hypothalamic expression levels are elevated in obesity and thus might contribute to the development of leptin resistance (166–168).

Members of the SOCS family are negative regulators of the JAK/STAT pathway. Leptin signaling *via* ObRb Y1138 and STAT3 rapidly induces SOCS3 expression (92, 166, 169). SOCS3 in turn will initiate a negative feedback loop by binding ObRb Y985, inhibiting further phosphorylation/activation of JAK2 (166, 170, 171). Immunohistochemical studies suggest that the ARC is selectively leptin resistant in DIO mice and that this may be caused by elevated SOCS3 expression (172). Neuron-wide deletion of SOCS3 leads to enhanced leptin-induced hypothalamic STAT3 phosphorylation and POMC induction, resulting in a more pronounced suppression of food intake, body weight loss, and an attenuation of diet-induced leptin resistance compared to wild-type mice (173). Specific deletion of SOCS3 in POMC neurons improves leptin sensitivity and glucose homeostasis (174). Consistently, increased SOCS3 expression in POMC neurons results in impaired STAT3 signaling with subsequent leptin resistance and obesity (175). Likewise, mice overexpressing a constitutively active version of STAT3 in POMC neurons show elevated SOCS3 expression and develop obesity as a result of hyperphagia and decreased POMC expression accompanied by central leptin resistance (176). A temporal and spatial pattern in leptin responsiveness was demonstrated before (177), and more recently, it was shown that SOCS3 activation in AgRP neurons precedes that of POMC and other hypothalamic neurons in the development of DIO (178).

Unexpectedly, SOCS3 upregulation in ObRb-expressing neurons does not lead to obesity, but rather a more lean phenotype (175). This may result from a compensatory increase in basal STAT3 expression and a corresponding increase in pSTAT3 levels after leptin treatment (175). However, inactivation of SOCS3 in ObRb-expressing cells protects mice from diet-induced insulin resistance, indicating that the regulation of leptin signaling by SOCS3 orchestrates diet-induced changes on glycemic control (179). Recently, ObRb-specific SOCS3^−/−^ mice were studied in fasting and refeeding conditions. These mice exhibit increased leptin sensitivity in the hypothalamus and show attenuated food intake and weight regain after 48 h of fasting by a lower transcription of orexigenic neuropeptides (180).

Protein tyrosine phosphatase 1B is localized to the cytoplasmic face of the ER and inhibits leptin signaling by binding and dephosphorylating JAK2 (181, 182). Mice with PTP1B-deficiency in the whole brain, ObRb expressing cells, or POMC neurons are lean, leptin hypersensitive, and display improved glucose homeostasis, supporting an antagonistic role for PTP1B in hypothalamic leptin signaling (121, 183, 184). Moreover, the impact of PTP1B on energy homeostasis seems to rely on hypothalamic ObRb signaling as the reduced adiposity seen after hypothalamic PTP1B depletion is reversed by the concomitant hypothalamic deletion of ObRb (185).

Tyrosine phosphatases (PTPs) such as TCPTP and RPTPϵ were demonstrated to modulate ObR signaling (186), since genetic ablation of neuronal TCPTP or whole-body RPTPϵ enhances leptin sensitivity (167, 186, 187). Other PTPs including SHP2 and PTEN may also have regulatory functions in ObRb signaling and were briefly discussed above.

### Hypothalamic Circuitry

Beyond leptin’s regulation of POMC and NPY/AgRP neurons on a transcriptional level, leptin can have a direct effect on these cells by altering neuronal firing (87). In addition, several lines of evidence have shown that the neuronal connectivity between specific subpopulations of hypothalamic and extra-hypothalamic neurons implicated in the regulation of energy balance is changing in response to high-fat diet (HFD) or metabolic hormones, such as leptin (188). For example, leptin treatment of *ob/ob* mice induces synaptic changes that precede the reduction in food intake and subsequent decrease in body weight (189). Although POMC and NPY/AgRP neurons in the ARC are considered to be the main targets of leptin, the majority of ObRb-expressing neurons lie outside the ARC in other central nervous system (CNS) regions known to modulate energy balance (88). This is underscored by the obese phenotype observed after deletion of ObRb in neurons, such as GABAergic neurons and NOS-1 (nitric oxide synthase-1) expressing neurons (190, 191). Growing evidence points to a critical role of astrocytes in orchestrating the hypothalamic response to metabolic cues by participating in processes of synaptic transmission and plasticity (192). Impaired ObR signaling in astrocytes leads to an altered glial morphology, increases the number of synapses onto POMC, and NPY/AgRP neurons and blunts leptin-induced anorexia (193).

### Defective Leptin Transport

To enter the brain, circulating leptin has to cross the BBB. During obesity, the cerebrospinal fluid/serum leptin ratio is decreased, indicating impairment of leptin transport (194, 195). Leptin is actively transported across the BBB in a saturable manner (196). The short ObR isoform, ObRa is believed to be implicated in this process (197, 198). However, more recent data contradict this theory. ObRa^−/−^ mice show only a small decrease in leptin responsiveness, suggesting that ObRa binding is not the only way by which leptin accesses the CNS (199). Moreover, pre-treatment with an ObR-neutralizing antibody, to directly examine the involvement of endothelial ObR in leptin transport, did not alter leptin transport in an *in vitro* BBB model (200). A role for megalin (or LRP2, low-density lipoprotein receptor-related protein-2) as a leptin transporter in the choroid plexus has been suggested (see also further) (200, 201).

The contribution of defective leptin transport to central leptin resistance remains unclear. Recently, it was demonstrated that the median eminence serves as the route through which leptin is transported into the hypothalamus, and that tanycytes act as a transport checkpoint. Peripherally administered leptin sequentially activates ObRb in median eminence tanycytes followed by neurons in the medio-basal hypothalamus (MBH) in a process that requires tanycytic ERK signaling (202). In obese mice (*db/db* or DIO) leptin accumulates in the median eminence and fails to reach the MBH, while triggering ERK signaling in tanycytes with epidermal growth factor (EGF) reestablishes leptin transport and its activation of MBH neurons (202).

### Leptin Receptor Trafficking

The amount of signaling-competent ObRb on the cell surface is determined by the balance between receptor synthesis, transport to the plasma membrane, internalization, recycling, degradation, and ectodomain shedding. At steady state, the ObRb is mainly retained in the Golgi complex or in a post-Golgi intracellular compartment, resulting in low levels at the cell surface, from where it undergoes constitutive removal *via* ligand-independent endocytosis leading to lysosomal degradation with no evidence of recycling (53). Feeding seems to control ObRb expression since ObRb levels in the ARC are increased after fasting and decreased by refeeding. Leptin increases ObRb expression in the ARC, but not after high-fat feeding (203). However, overexpression of ObRb in POMC neurons renders mice more susceptible to DIO, further underlining the importance of correct ObRb expression (204).

Endospanin 1 [also known as Ob-RGRP (ObR gene-related protein) or LEPROT (leptin receptor overlapping transcript)], whose expression is genetically linked to the ObRb transcript, negatively controls ObRb cell surface expression (205). Endospanin 1 interacts with ObRb and targets ObRb from endosomes to lysosomes, thereby increasing its degradation (206). Hence, endospanin 1 silencing in the ARC is sufficient to prevent or reverse the development of obesity after high-fat diet in lean or fully obese mice, respectively (205, 207).

Our group identified the E3 ubiquitin ligase RNF41 (Ring Finger Protein 41) as an interaction partner of the ObRb complex (17). RNF41 acts as a key regulator of basal cytokine receptor trafficking, proteolytic processing, and signaling. RNF41 controls the constitutive intracellular trafficking of the ObRb, by preventing its lysosomal receptor degradation, and concomitantly enhancing receptor ectodomain shedding by the metalloprotease ADAM10 (17). We further demonstrated that this results from RNF41-dependent ubiquitination and suppression of the deubiquitinating enzyme USP8, which abrogates ESCRT-0 functionality and accounts for the rerouting of cytokine receptors (208).

Accumulating evidence suggests that the neuronal cilia basal body complex acts as a platform for ObRb signaling. Neuronal cilia lengths were selectively reduced in the hypothalamus of obese mice with leptin deficiency and leptin resistance, while treatment of hypothalamic neurons with leptin-stimulated cilia assembly *via* inhibition of PTEN and glycogen synthase kinase 3β (GSK3β) (209, 210). Moreover, mice with short hypothalamic cilia exhibit increased food intake, decreased energy expenditure, and attenuated anorectic responses to leptin, which indicates that leptin-induced cilia assembly is essential for sensing leptin by hypothalamic neurons (209). Bardet–Biedl syndrome (BBS) proteins form the stable BBSome complex, which mediates protein trafficking to the ciliary membrane (211). The BBSome influences energy homeostasis through the control of ObRb transport to the cell surface expression as targeted disruption of the BBSome by deleting BBS1 in ObRb-expressing cells causes obesity in mice (212, 213). Suppression of another ciliary gene, retina pigmentosa GTPase regulator-interacting protein-1 (RPGRIP1L), in neuronal cultures decreases localization of ObRb near the cilium and activation of the downstream signaling cascade (214). Like BBS proteins, RPGRIP1L interacts with the ObRb and mediates its trafficking to the periciliary area (215). Mice hypomorphic for *RPGRP1L* exhibit hyperphagic obesity as the result of diminished leptin sensitivity in ObRb-expressing neurons (216).

### ER Stress and Inflammation

Several studies have provided evidence that ER stress and the activated adaptive unfolded protein response (UPR) impair leptin signaling and are highly increased in hypothalamic neurons in the context of obesity (217, 218). Reducing hypothalamic ER stress by chemical and natural chaperones can re-establish leptin sensitivity (217, 219, 220). Xbp1s (spliced X-box binding protein 1) is one of the ER-stress-induced genes and neuron-specific Xbp1^−/−^ mice have ER stress, severe hyperleptinemia, leptin resistance, and obesity (217). In contrast, induction of Xbp1s in POMC neurons alone is sufficient to protect against DIO and to improve leptin sensitivity by suppressing SOCS3 and PTP1B, even in the presence of strong ER stress activators (221). Several mechanisms are suggested to bridge HFD-induced ER stress to impaired ObR signaling (222). Overnutrition atypically activates IKKβ-NFκB signaling in the hypothalamus through ER stress responses, which implies a connection between ER stress and hypothalamic inflammation (218). Indeed, obesity seems to be associated with low-grade chronic inflammation (223). Saturated fatty acids, which are elevated in obesity, are able to bind and activate toll-like receptor 4 (TLR4) and inhibition of TLR4 or neuronal deletion of the TLR adaptor molecule MyD88 protects from HFD-induced leptin resistance and obesity (224–226). Inhibition of IKKϵ, a downstream molecule of NFκB signaling, reduces leptin resistance by restoring JAK2-STAT3 and PI3K signaling in the hypothalamus of HFD-fed mice (227).

It was recently shown that disruption of mitochondria–ER contacts may also contribute to leptin resistance development in POMC neurons. Mitochondrial-ER contacts are decreased in POMC neurons of mice receiving HFD (228). Furthermore, deletion of PPARγ in POMC neurons enhances mitochondrial-ER interactions and sensitizes POMC neurons to leptin during HFD (229). On the other hand, mice with a POMC-specific deletion of MFN2 (mitofusin 2), a key protein for mitochondrial fusion and the formation of ER–mitochondria contacts, display a loss of these interactions, defective POMC processing, ER stress-induced leptin resistance, and obesity (228). In contrast, ablation of mitofusins in NPY/AgRP neurons disrupts mitochondrial fusion without inducing ER stress and alleviates HFD-induced obesity (230). These findings indicate the importance of mitochondrial dynamics in hypothalamic neurons during the establishment of DIO.

### Redefine the View on “Leptin Resistance”?

Recently, Ottaway et al. reported that central or peripheral administration of an ObR antagonist induces comparable changes in food intake, body weight, and hypothalamic POMC and SOCS3 expression in lean and DIO mice, illustrating that endogenous ObR signaling may not be reduced in the context of DIO, thus challenging the established concept of leptin resistance under dietary-induced conditions (231). More efforts are required to further comprehend the link between the different mechanisms mentioned above, and the precise sequence of appearance of the alterations, discriminating between causes and consequences (232). Moreover, many other nutrient signals, such as insulin and ghrelin, contribute to the control of energy homeostasis. Cross-talk and redundancy between these signals complicate the precise assessment of the contribution of leptin-mediated signaling in the context of leptin resistance and obesity.

## Leptin Signaling Beyond the ObR: Cross-Talk at the Cellular Surface

Several independent lines of evidence suggest that leptin signaling in the brain or the periphery can be different. In a first example, Nizard and colleagues reported the pregnancy of a morbidly obese patient with a rare ObR mutation (233). Normally, such loss-of-function mutations have been linked to infertility in humans and rodents (29, 234). Despite neonatal hypoglycemia, the child’s growth and development have been normal (233). Second, a serine to leucine mutation on position 72 in leptin hampers the secretion, but not the expression of leptin in adipose tissue in a 14-year-old child of non-obese Austrian parents (235). The child showed signs of a hypogonadotropic hypogonadism, but in contrast to previous studies only mild obesity and a normal T cell responsiveness. Third, a spontaneous splice-mutation causes the deletion of the complete IGD domain in *fatt/fatt* mice (236). These animals are hyperphagic and obese, but show minimal changes in size and cellularity of the thymus and respond comparable to wild-type animals to concavalin A in a model for autoimmune hepatitis. Finally, treatment of healthy mice with a IGD-specific neutralizing nanobody induced clear weight gain and hyperinsulinemia, but failed to block development of experimentally induced autoimmune multiple sclerosis, arthritis, and hepatitis (236).

Cross-talk with other (cytokine) receptors could explain this observed uncoupling of leptin’s central and peripheral functions. In a simplistic view, a cytokine triggers intracellular signaling by binding and activation of a cognate homo- or heteromeric receptor pair. However, when combined, cytokines, hormones, and other stimuli might have additive, synergistic, or antagonistic effects. This so-called “cross-talk” does not only occur at the level of signaling pathways but also at the cell surface between cytokines, hormones, and their receptors. The process that a certain cytokine activates another receptor complex is called cross-activation. At the moment, it is mostly unclear how a cytokine discriminates between different options, but cell-specific co-expression is likely a determining factor.

At the cell surface, the leptin:ObR system can interact with the epidermal growth factor receptor (EGFR), estrogen receptor alpha (ERα), insulin-like growth factor I receptor (IGF-IR), lipoprotein receptor-related protein 1 and 2 (LRP1 and LRP2), and vascular endothelial growth factor receptor (VEGFR). Studies describing ObR cross-talk and cross-activation are summarized in Table 1.

**Table 1:** **Overview of studies describing ObR cross-talk and/or cross-activation (see text for more details)**.

	Background	Model	Effects	Reference
Epidermal growth factor receptor (EGFR)	*In vitro*	Gastric cancer cells (MKN28 and MKN74)	Leptin-induced EGFR phosphorylation	(237)
			EGFR inhibitor AG1478 blocks leptin-induced JAK2 and ERK1/2 activation	
	*In vitro*	Human breast cancer cells (MCF7 and MDA-MB-231)	Leptin induces clonogenicity, anchorage-independent growth, migration, and	(238)
			upregulation of survivin and Notch-I expression and EGFR phosphorylation	
	*In vitro*	Esophageal adenocarcinoma cells (OE33, OE19, BIC-1, and FLO)	Leptin induces proliferation, inhibits apoptosis, and enhances EGFR and ERK1/2 phosphorylation	(239, 240)
			EGFR inhibitors AG1478, and PD98059 block these effects	
	*In vitro*	Rat aortic smooth muscle cells	Leptin induces EGFR and ERK1/2 phosphorylation and endothelia-1 expression	(241)
			EGFR inhibitor AG1478 blocks leptin-induced ERK1/2 phosphorylation and endothelia expression	
	*In vitro*	Gastric mucosal cells	Leptin induces EGFR and cPLA2 phosphorylation and protects against ethanol cytotoxicity	(242)
			EGFR inhibitor AG1478 blocks phosphorylation events and the protection	
	*In vitro*	Rat sublingual salivary gland acinar cells	Leptin induces EGFR and cPLA2 phosphorylation and protects against ethanol cytotoxicity	(243)
			EGFR inhibitor AG1478 blocks phosphorylation events and the protection	
	*In vitro*	Transfected Hek293T cells	Both long and short ObR forms trans-phosphorylate and activate EGFR	(244)
	*In vivo*	Rat kidneys	Leptin induces Na^+^, K^+^-ATPase activation, H_2_O_2_ excretion, and ERK1/2 phosphorylation	(245)
			EGFR inhibitors, AG1478 and PD98059, block these effects	

ERα	*In vitro*	Human breast cancer cells (MCF7 and MDA-MB-231)	ERα downregulation abolishes leptin-induced STAT3 phosphorylation independent of ERα ligands	(246)
			ERα binds to JAK2 and STAT3 thereby increasing kinase activity and cell viability	
	*In vitro*	Human breast cancer cells MCF7	Leptin enhances aromatase expression *via* AP-1 and STAT3 and ERK1/2 dependent	(247)
	*In vitro*	Human breast cancer cells MCF7 and transfected HeLa cells	Leptin induces ERα nuclear localization and controls ERα expression on mRNA and protein level	(248)
			Leptin treatment potentiates estradiol-induced activation of ERα	
	*In vitro*	Human breast cancer cells MCF7	Leptin increases ERα expression	(249)
	*Ex vivo*	Breast cancer patients	Significant correlation between ObR and ERα levels	
	*In vivo*	Mouse MCF7 xenografted nude mice	Leptin increases ERα and decreases ERβ levels on mRNA and protein level	(250)

Insulin-like growth factor I receptor (IGF-IR)	*In vitro*	Human breast cancer cell lines (MDA-MB-231, BT474, and SKBR3)	ObR and IGF-IR can be co-immunoprecipitated Unidirectional: IGF-I induced ObR phosphorylation depends on IGF-IR kinase activity	(251)
	*In vitro*	Human breast cancer cells (MCF7, MDA-MB-231, and MDA-MB-468)	Bidirectional: IGF-I induced ObR phosphorylation, leptin IGF-IR phosphorylation Leptin and IGF-I co-treatment synergistically cross-activated EGFR	(252)
			EGFR cross-activation promotes metastatic properties, invasion, and migration	

LRP1	*In vivo*	*Lrp1* forebrain knockout mice	Conditional deletion of LRP1 decreases leptin signaling and results in an obese phenotype	(253)
			LRP1 interacts with ObR and is required for STAT3 phosphorylation	

LRP2	*In vivo* and *in vitro*	Mice, rats, Rhesus Macaques, and yolk sac L2 cells	PET imaging illustrates that leptin is rapidly taken up by LRP2 in the renal tubules Leptin uptake is also mediated by LRP2 in L2 cells	(254)
	*In vivo*	Wistar rats	LRP2 mediates transport of leptin through the choroid plexus	(255)
			Choroid plexus LRP2 expression correlates with leptin uptake	
	*In vivo* and *in vitro*	Wistar rats and yolk sac L2 cells	Labeled leptin in rats is filtered by glomeruli and internalized by proximal convoluted tubules LRP2 binds leptin in a Ca^2+^-dependent manner and mediates internalization and degradation	(256)

Vascular endothelial growth factor receptor (VEGFR)	*In vitro*	Human umbilical vein endothelial cells (HUVEC)	Leptin induces HUVEC proliferation, cyclo-oxygenase-2 expression, and VEGFR2 phosphorylation	(257)
			Inhibition of VEGFR-2 kinase activity blocks the leptin-induced effects	
	*In vitro*	Human umbilical vein and porcine aortic endothelial cells	Leptin trans-phosphorylates VEGFR-1 and VEGFR-2	(258)
			Leptin induces Notch signaling, proliferation, and tube formation in these endothelial cells	
			Effects can be blocked by Notch and VEGFR inhibitors	

### Epidermal Growth Factor Receptor

The EGFR is a single membrane-spanning receptor with a cytoplasmic tyrosine kinase domain. This receptor can be activated not only by its “natural” ligands, including EGF, transforming growth factor-α (TGF-α), heparin-binding EGF-like growth factor, amphiregulin, and epiregulin but also by cross-activation by G-protein-coupled receptors, the tumor necrosis factor receptor, or IGF-IR [reviewed in Ref. (259)]. ObR:EGFR cross-talk and cross-activation is not restricted to cancer cell lines, but could also be demonstrated in muscle, salivary gland, and mucosal cells and in rat kidneys (see Table 1). The cross-talk often involves the activity of ERK and Src kinases and can be blocked by EGFR kinase inhibitors. Both ObR long and short forms are able to phosphorylate the EGFR, suggesting that this cross-activation is independent of downstream ObR signaling (244). It was suggested that this cross-talk depends on the proteolytic release of EGFR ligands as broad-spectrum matrix metalloproteinase inhibitor (e.g., GM6001) blocks the leptin-induced effects. However, this pathway likely does not explain the rapid (within 5 min) and transient increase in EGFR tyrosine phosphorylation by leptin in, for example, rat aortic smooth muscle cells (241).

### ERα

A significant co-expression between ObR and ERα and a functional cross-talk between the leptin and estrogen signaling networks are associated with breast tumor progression [reviewed in Ref. (260)]. Leptin not only controls ERα and aromatase mRNA expression both *in vitro* and *in vivo* and in an ERK and STAT3-dependent manner (247, 249, 250), one report shows that it can directly cross-activate ERα in the absence of cognate ligand (248).

### Insulin-Like Growth Factor I Receptor

Insulin-like growth factor I receptor is an α_2_: β_2_ disulfide-linked receptor that shares more than 50% of overall sequence homology with the IR. It is overexpressed in 50% of primary breast tumors compared with normal tissue (261), and IGF-IR inhibition reduces cancer growth and metastasis *in vivo* (262). Saxena et al. and Ozbay et al. independently demonstrated the ObR:IGF-IR cross-talk in breast cancer cells: both IGF-I and leptin phosphorylate the reciprocal receptor, ObR and IGF-IR can be co-immunoprecipitated, and combined treatment increased proliferation, invasion, and migration of breast cancer cells (251, 252).

### LRP1/LRP2

LRP1 is highly expressed in neurons of the CNS and plays a role in lipoprotein metabolism, neurotransmission, synaptic plasticity, and cell survival (263). Liu et al. showed that conditional deletion of LRP1 results in an obese phenotype, characterized by increased food intake, decreased energy expenditure, and hampered leptin signaling. LRP1 directly interacts with ObR and is necessary for STAT3 activation (253).

LRP2 or megalin is a large glycoprotein abundantly expressed at the apical membranes of proximal tubule cells that reabsorb and metabolize proteins filtered by glomeruli in the kidney (264). A role of LRP2 in leptin clearance (254, 256), and/or transport over the BBB has been suggested (see above) (255).

### Vascular Endothelial Growth Factor Receptor

Vascular endothelial growth factor receptor and its receptor play a crucial role in the angiogenic process in physiologic and pathological scenarios (265). In human umbilical vein endothelial cells and porcine endothelial cells, leptin trans-phosphorylates VEGFR-1 and VEGFR-2, induces cyclo-oxygenase-2 and Notch signaling, proliferation and tube formation (257, 258).

## Leptin Signaling Beyond the ObR: Cross-Talk Between Downstream Signal Cascades

Cross-talk does not only occur at the receptor level (receptor co-complexes, cross-activation) but also more downstream at the level of activated kinases and transcription factors or due to leptin-dependent expression of other cytokines.

### Leptin–Insulin

Both leptin and insulin elicit strong anorectic responses within the ARC and their central administration, which mimics a state of energy surplus, inhibits food intake, and decreases body weight (266). One prevailing view is that different POMC neurons exist and that leptin and insulin may act on distinct POMC neuronal subsets (127). However, many studies demonstrate cross-talk between leptin and insulin signaling at many levels [reviewed in Ref. (267)]. The insulin pathway, after insulin binding to the IR and activation of the IRS proteins, converges with the leptin pathway at the same point, the activation of PI3K, to modulate body weight and glucose homeostasis (268). Also, both leptin and insulin regulate the AMPK pathway in the hypothalamus and inhibit AMPK activation and its downstream targets (153, 154). Recently, it was shown that insulin can potentiate leptin-induced STAT3 activation by the induction of GRP78 (glucose-regulated protein 78), a UPR chaperone that is required to maintain ER capacity and protect against ER stress (269). Strikingly, ICV administration of leptin in mice devoid of insulin and lacking ObRb demonstrates that concomitant re-expression of ObRb only in hypothalamic GABAergic and POMC neurons is sufficient to fully mediate the anti-diabetic actions of leptin in insulin deficiency (270). Likewise, cellular insulin resistance disrupts leptin-mediated control of neuronal signaling and transcription (271).

### NILCO (Notch, IL-1, and Leptin Cross-talk Outcome)

Functional cross-talk between leptin, IL-1, and Notch signaling (NILCO) is found in breast cancer cells and could represent the integration of developmental, pro-inflammatory, and pro-angiogenic signals which are critical for leptin-induced breast cancer cell proliferation/migration and tumor angiogenesis. Inhibition of leptin signaling significantly reduces the establishment and growth of breast cancer and simultaneously decreases the levels of VEGF/VEGFR2, IL-1 and Notch (272–274). Therefore, inhibition of leptin–cytokine cross-talk might serve as a preventative or adjuvant measure to target breast cancer, particularly in obese women.

### Cross-talk with Pro-inflammatory Cytokines

Pro-inflammatory cytokines may have differential roles in hypothalamic leptin signaling. As mentioned before, overnutrition leads to increased expression of pro-inflammatory cytokines, such as tumor necrosis factor-α (TNF-α), IL-1β, and IL-6 (223, 275). Lack of TNFR1 mitigates leptin resistance under HFD conditions, while ICV co-injection of TNFα partially blocks leptin’s anorexigenic effect through the inhibition of PI3K–Foxo1 signaling (276, 277). Conversely, central injection of an IL-1R antagonist or IL-1R1 knock-down blunts the suppression of food intake in response to leptin (278). Likewise, central infusion of IL-6 enhances hypothalamic STAT3 phosphorylation and suppresses hypothalamic IKKβ activation and hyperphagia in DIO (279).

### Toll-Like Receptor 4

Toll-like receptor 4 and ObRb activation seem to converge at a common signaling point in the hypothalamus. LPS, a ligand of TLR4, stimulates PI3K and STAT3 signaling pathways in cells expressing ObRb. Genetic deletion of the PI3K p110β catalytic subunit in ObRb-expressing cells leads to blunted suppression of food intake by LPS which demonstrates that lowered food intake during an inflammatory challenge depends on the PI3K pathway activated by cytokines and leptin in hypothalamic neurons (280). Moreover, NFκB signaling in POMC neurons is activated by leptin and mediates leptin-stimulated POMC transcription, indicating that hypothalamic NFκB also serves as a downstream transcription factor of the ObRb (281). Increased hypothalamic POMC promoter methylation in mice with DIO limits NFκB binding, which limits the ability of leptin to increase POMC expression (282).

## Future Perspectives

It is more than 20 years ago that leptin and its receptor have been identified as key regulators of body weight and energy homeostasis. However, the hormone mostly failed in the clinic to treat obesity due to the fact that obese people are almost always hyperleptinemic and resistant to leptin. The observation that leptin has functions in immunity, hematopoiesis, angiogenesis, reproduction, and BP, and is involved in the pathology of, e.g., autoimmune diseases and cancers, reopened the interest in leptin and ObR-based therapeutics. Current strategies, including leptin mutants, leptin peptide antagonists, neutralizing antibodies, and soluble receptors, were shown to be effective in the treatment of several autoimmune diseases and in some cancer models [reviewed in Ref. (283)]. Their clinical application is, however, hampered by the unwanted weight-gain (10–15% per week in rodent models) upon treatment.

The ability of the ObR to interact with other receptor systems at the receptor level or intracellulary illustrates that the leptin receptor complex may be “heavier” than expected. This creates new exciting opportunities including the possibility to uncouple leptin’s central role in weight regulation and its peripheral functions. More in-depth insights in these complex leptin/ObR activation and downstream signaling mechanisms may ultimately allow the design of selective antagonists.

## Author Note

The authors apologize to their colleagues that space limitations did not allow us to cite all the relevant literature.

## Author Contributions

JW and LZ wrote the review, while JT was responsible for revising.

## Conflict of Interest Statement

The authors declare that the research was conducted in the absence of any commercial or financial relationships that could be construed as a potential conflict of interest.

## Abbreviations

ACC, acetyl-CoA carboxylase; AgRP, agouti-related peptide; AMPK, adenosine monophosphate-activated protein kinase; α-MSH, α-melanocyte stimulating hormone; AR, amphiregulin; ARC, arcuate nucleus; BBB, blood–brain barrier; BBS, Bardet–Biedl syndrome; BP, blood pressure; BRET, bioluminescence resonance energy transfer; CNS, central nervous system; CRH, cytokine receptor homology domain; EGF, epidermal growth factor; EGFR, EGF receptor; EPR, epiregulin; ER, endoplasmic reticulum; ERK, extracellular signal-related kinase; FN III, fibronectin type III; Foxo1, forkhead box O1; FRET, fluorescence resonance energy transfer; GABA, γ-aminobutyric acid; G-CSF, granulocyte-colony stimulating factor; Grb2, growth factor receptor-bound protein 2; GRP78, glucose-regulated protein 78; GSK3β, glycogen synthase kinase 3β; HB-EGF, heparin-binding EGF-like growth factor; HFD, high-fat diet; HUVEC, human umbilical vein endothelial cell; ICV, intracerebroventricular; IGD, immunoglobulin-like domain; IGF-IR, insulin-like growth factor I receptor; IL, interleukin; IR, insulin receptor; IRS, insulin receptor substrate; JAK, Janus kinase; LEPROT, leptin receptor overlapping transcript; LRP, lipoprotein receptor-related protein; MALLS, multi-angle laser light scattering; MAPK, mitogen-activated protein kinase; MBH, medio-basal hypothalamus; MFN2, mitofusin 2; mTOR, mammalian target of rapamycin; NOS-1, nitric oxide synthase-1; NPY, neuropeptide Y; NTD, N-terminal domain; ObR, leptin receptor; PDE3B, phosphodiesterase 3B; PDK1, 3-phosphoinositide-dependent protein kinase; PI3K, phosphatidylinositol 3 kinase; PIP_3_, phosphatidylinositol 3,4,5-triphosphate; POMC, pro-opiomelanocortin; PRMT2, protein arginine *N*-methyltransferase 2; PTEN, tumor suppressor phosphatase and tensin homology; PTP, tyrosine phosphatase; PTP1B, protein tyrosine phosphatase 1B; RNF41, ring finger protein 41; RPGRIP1L, retina pigmentosa GTPase regulator-interacting protein-1; S6K, S6 kinase; SAXS, small-angle X-ray scattering; SHP2, SH2-containing protein tyrosine phosphatase 2; SOCS, suppressor of cytokine signaling; STAT, signal transducer and activator of transcription; TGF-α, transforming growth factor-α; TLR, toll-like receptor; TNF-α, tumor necrosis factor-α; UPR, unfolded protein response; VEGF, vascular endothelial growth factor receptor; Xbp1s, spliced X-box binding protein 1.
